# Healthcare Providers’ Perspectives on the Communication Challenges When Discussing Palliative Sedation: A Qualitative Study Across Eight European Countries

**DOI:** 10.3390/jcm14186653

**Published:** 2025-09-21

**Authors:** Éva Pozsgai, Csilla Busa, Holger Brunsch, Michael Van der Elst, Sheila Payne, Nancy Preston, Ian Koper, Jeroen Hasselaar, Rocio Roji, Claudio Adile, Daniela Mosoiu, Camelia Ancuta, Ágnes Csikós

**Affiliations:** 1 Department of Primary Health Care, University of Pécs Medical School, Rákóczi Street 2, 7623 Pécs, Hungary; 2 Department of Public Health Medicine, University of Pécs Medical School, Szigeti Street 12, 7624 Pécs, Hungary; 3 Department of Palliative Medicine, University Hospital Bonn, Venusberg-Campus 1, 53127 Bonn, Germany; 4 Laboratory for Experimental Radiotherapy, Department of Oncology, University of Leuven, Herestraat 49, 3000 Leuven, Belgium; 5 International Observatory on End of Life Care, Faculty of Health and Medicine, Lancaster University, Lancaster LA1 4AT, UK; 6 Department of Primary and Community Care, Radboud University Medical Centre, Geert Grooteplein 21, 6525 EZ Nijmegen, The Netherlands; 7 IdISNA—Instituto de Investigación Sanitaria de Navarra, 31008 Pamplona, Navarra, Spain; 8 Clínica Universidad de Navarra, 31008 Pamplona, Navarra, Spain; 9 La Maddalena Cancer Center, Via San Lorenzo 312, 90146 Palermo, Italy; 10 Medical Faculty Brasov, Transilvania University, Balcescu Street, 500036 Brasov, Romania; 11 Hospice Casa Sperantei, Brasov Sitei Street 17a, 500074 Brasov, Romania

**Keywords:** palliative sedation, communication, palliative care, moral case deliberation, qualitative research, death, dying

## Abstract

**Background/Objectives:** Studies have shown that healthcare providers struggle to discuss difficult end-of-life issues, including palliative sedation (PS), with patients and relatives. This qualitative study aimed to evaluate communication challenges related to PS among healthcare providers in eight European countries. **Methods:** In each country, two clinical settings providing palliative care were selected. Two moral case deliberation (MCD) sessions were conducted, each with 3 to 9 palliative healthcare professionals (HCPs). They discussed patient cases involving PS and refractory symptom management. Sessions were audio-recorded, transcribed, anonymized, and analyzed using a framework analysis. **Results:** Key issues included core communication values—open, empathetic, and honest dialogue—which were consistent across countries but varied in practice. In The Netherlands, Germany, Belgium, and the UK, communication prioritized patient autonomy through timely discussions and family dialogue. In Spain and Italy, family-centered communication approaches predominated, while in Romania and Hungary, tendencies for selective disclosure were also evident, along with delegated decision-making and complex family dynamics. Certain challenges reflected professional experience rather than national culture. Nurses mediated across contexts, while terminology surrounding palliative sedation remained a source of ambiguity. **Conclusions:** This is the first study to present HCPs’ perceptions of communication issues related to PS across Europe. Despite variations between settings, consistently open dialogue among patients, families, and HCPs emerged as the most valued element. These findings highlight the need to better understand how end-of-life communication about PS varies in practice and underscore the importance of considering healthcare providers’ real-world experiences to improve communication with patients and families.

## 1. Introduction

Healthcare professionals can only provide appropriate palliative care if they understand the needs and preferences of the individuals involved [1,2,3]. Achieving this requires continuous and effective communication between healthcare providers, patients, and their families, which is essential for delivering quality palliative care [4].

Several studies have indicated that physicians still struggle to discuss prognosis and palliative care issues with patients facing advanced illness, particularly concerning topics such as palliative sedation [5]. Consequently, the needs, objectives, and wishes regarding care are often not discussed promptly and adequately with the patients and their families [6,7,8]. This results in physicians frequently responding reactively rather than proactively [8]. Many aspects of palliative sedation, particularly the specifics of communication related to it, remain unexplored, highlighting a gap in data on communication issues.

Recent international literature highlights the critical but distinct roles of other healthcare professionals in end-of-life communication. Nurses, who often provide the majority of bedside care, spend the most time with patients and families and frequently navigate emotionally and ethically complex situations [9,10]. A study conducted in Poland with master’s students in nursing and midwifery highlighted the most debated ethical and legal dilemmas, along with the factors shaping students’ perspectives, suggesting that greater attention should be given to these issues in their training programs [10]. Other multidisciplinary team members—including social workers, psychologists, and chaplains—also contribute to communication and decision-making in end-of-life discussions and palliative sedation. Social workers and psychologists support families in coping with distress and understanding medical information, while chaplains provide spiritual guidance and address existential concerns [11]. Interdisciplinary collaboration has been shown to ensure consistent, culturally sensitive communication, reduce moral distress, and enhance patient-centered care [12].

Palliative sedation, or sometimes simply referred to as sedation, is a medical intervention used in the final phase of life to alleviate intractable suffering in patients with life-limiting illnesses by deliberately reducing consciousness through the controlled administration of medication [13]. This procedure is considered when severe symptoms associated with the patient’s disease—such as pain, delirium, and dyspnea—become refractory, exhausting all treatment options, and causing immense suffering and distress for the patient [14].

Although palliative sedation is used only when indicated and as a last resort, it remains one of the most often debated medical treatments [15,16]. This procedure raises ethical questions, particularly regarding the depth and duration of sedation [5], and its application in cases of not only physical but also existential suffering [15]. Additionally, the terminology can be confusing and often leads to misunderstandings among patients and families, who may misinterpret palliative sedation as physician-assisted suicide or euthanasia [5,17,18]. The cultural and religious backgrounds of those involved can further influence these perceptions [19], for example, the value of being conscious until the last moment of life.

Clinical guidelines for palliative sedation are crucial in aiding decision-making [15,17]. However, most recommendations have focused on the clinical aspects of the procedure, with less emphasis on communication-related issues [15]. Along with clinical competencies, specific communication skills are essential for healthcare professionals caring for patients being considered for palliative sedation [20,21]. Although there are new clinical guidelines, they have only recently been published, so it is too early to have a widespread impact [18].

To address these challenges related to palliative sedation, healthcare professionals in some countries have adopted the practice of holding moral case deliberation (MCD) sessions in multidisciplinary teams to address care issues that raise ethical issues [16,20,22]. These sessions help professionals navigate difficult ethical issues by identifying key values and considering all stakeholders’ viewpoints, values, and norms. This approach relieves distress for healthcare professionals, patients, and family members alike [23,24].

Investigating practices across countries is essential, as communication around palliative sedation is shaped by broader healthcare systems, cultural norms, and ethical frameworks. A multi-country perspective makes it possible to identify both shared communication challenges that transcend national boundaries and context-specific practices shaped by local settings. This dual focus—on recurring patterns and local differences—can inform culturally sensitive practice, guide education and guideline development, and help clinicians adapt communication strategies to diverse healthcare contexts.

Consequently, as part of a larger European Union project examining palliative sedation in clinical practice, we aimed to investigate the primary communication issues reported by healthcare providers during MCD sessions across eight European countries. Our objective was to provide a descriptive perspective on these challenges, taking into account diverse healthcare and cultural contexts.

## 2. Methods

### 2.1. Study Design

This study was part of the Palliative Sedation project, a five-year initiative involving investigators from eight European countries (Belgium, Germany, Hungary, Italy, The Netherlands, Romania, Spain, and the United Kingdom) dedicated to exploring the application of palliative sedation (https://palliativesedation.eu, accessed on 04.04.2025). Ethical approval was obtained prior to the study’s commencement, and financial support was provided by the European Union’s Horizon 2020 research and innovation program under grant agreement No. 825700.

This study aimed to describe the perceptions of healthcare providers regarding communication issues surrounding palliative sedation and the ethical issues it raised.

### 2.2. Settings and Participants

The study involved two clinical sites in each country, selected on the following inclusion criteria: (1) the site provided care for patients with palliative care needs, refractory symptoms, and/or offered palliative sedation; (2) care was delivered by multidisciplinary teams (minimum requirements: a physician, a nurse, and other relevant palliative care staff); and (3) the site offered inpatient care.

Participants were included based on these criteria: (1) experience in professional care for patients with palliative care needs and/or refractory symptoms; (2) membership in a multidisciplinary care team; and (3) ability to communicate in the national language of the country.

Senior clinicians at each site were responsible for identifying and enlisting healthcare providers to participate in the MCD sessions. Invitations, along with study information sheets, were sent to potential participants via email. Informed consent was gained from all participants prior to commencing the MCD session.

The selected clinical sites provided care for patients with palliative care needs and/or refractory symptoms, predominantly located in palliative or hospice care units (as detailed in Table 1). A total of 32 MCD sessions were conducted across these sites, involving physicians and nurses in all sessions, supplemented with psychologists, physical therapists, counsellors, social workers, and faith workers, depending on the site. Overall, 231 healthcare providers participated in the 32 MCD sessions, with an average attendance of seven individuals per session, ranging between 3 and 9 participants.

Table 1 shows the characteristics of the clinical sites and the number of participants in each MCD session.

### 2.3. Case Preparation for MCD Sessions and Data Collection

During the MCD sessions, relevant patient cases related to palliative sedation and/or refractory symptom management, as agreed upon by the healthcare provider team at each site, were discussed.

Inclusion criteria for the cases were (i) patients with refractory symptoms, (ii) consideration of palliative sedation, and (iii) cases presenting significant moral dilemmas. Exclusion criteria were cases focused solely on medical or technical aspects without an ethical dimension.

Case narratives included the patient’s personal and family context, and were limited to approximately one page, supplemented by a half-page summary of relatives’ perspectives. To ensure quality and consistency, case preparation followed a multi-step review process involving draft development, revision, and final approval in collaboration between the healthcare provider team and the research team at each site. Prior to group meetings, all participants received the case description by email. During the session, designated colleagues were responsible for presenting the case and articulating family viewpoints, particularly when perspectives diverged.

The MCD sessions were conducted from October 2020 to March 2022, facilitated by researchers who received training from an experienced ethicist on the facilitation process. Each session lasted an average of 82 min (ranging from 55 to 115 min) and included at least one observing researcher in addition to the facilitator.

All MCD sessions followed a consistent structure, as previously described by Stolper et al. [23].

### 2.4. Data Analysis

Data analysis was performed using the seven-stage framework method [25].

Each MCD session was audio recorded with the participants’ consent, plus additional field notes were taken. The recordings were transcribed, anonymized, and reviewed prior to analysis.

National transcripts from three countries (the UK, Hungary, and Romania) were examined by researchers to familiarize themselves with the content, a process that contributed to the development of a shared coding framework.

Professional translators translated the Romanian and Hungarian transcripts into English. Senior researchers from the UK, Romania, and Hungary used sensitizing concepts from the study protocol to develop an initial set of codes. These initial codes were piloted and refined using one transcript from each country.

This resulted in a preliminary codebook that incorporated both sensitizing concepts and inductively identified codes.

The framework was then systematically applied to all transcripts from all countries using the established codes.

The analytical framework was then systematically applied to all transcripts using the established codebook.

Researchers from all countries participated in joint, practice-oriented sessions to align their understanding and application of the codes, ensuring consistency across the team. NVivo 12 qualitative data analysis software was used to support coding and management of the data.

Each researcher applied the preliminary codebook to transcripts in their native language. Ongoing discussions with the wider research team allowed for the identification of emerging concepts, clarification of code definitions, and integration of additional codes where necessary. Minor revisions were made to the codebook based on these team discussions.

The finalized international codebook was used to complete the coding process and to support structured, cross-national analysis using the framework matrix. Summarized data were charted into matrices organized by country and thematic area, allowing for comparison across settings. Country-specific reports were compiled in English and illustrated with selected participant quotes.

The final stage of the framework method—data interpretation—involved collaborative analysis across the research team to generate explanatory insights and interpret thematic patterns.

A second round of framework analysis was conducted on the eight country reports using the final codebook, focusing on identifying commonalities and differences in how issues regarding palliative sedation were understood and managed.

Throughout all stages of analysis, regular online meetings were held with researchers from the eight participating countries to facilitate shared understanding and reflexivity.

Illustrative quotes were selected by the national research teams based on the coded data, ensuring alignment with the analytic themes.

It is important to note that the perceptions expressed by HCPs in various settings do not necessarily reflect or represent the views of all HCPs across the entire country. Therefore, any mention of a country within the text refers to an MCD session in that respective country and not the practices of the country as a whole.

## 3. Results

The main communication issues mentioned during the MCDs related to palliative sedation can be categorized into general issues and issues specific to interactions between HCPs and the patient, HCPs and the family, and between the patient and the family. These main categories and key issues discussed across the eight European country settings are summarized in Table 2 and described in detail below.

### 3.1. General Communication Issues Related to Palliative Sedation

All HCP teams emphasized the importance of empathic, clear, open, and honest communication, viewing it as fundamental to establishing trust among the HCP team, patients, and family members when discussing palliative sedation.

One HCP in Spain mentioned that without tailored communication for patients and their families, trust can be easily lost:

*“Regarding the criteria, that in the end it is not an à la carte medicine. For example, the family may have a lack of trust in the professional team and if you don’t respond to their requests that they are suffering. That is, they may stop trusting you as a person who heals or helps.”*

(Doctor 3, Spain)

Patients and families have the right to be informed, and the flow of information among HCPs, patients, and family members is crucial, as emphasized by HCPs in the UK, Germany, and Belgium.

*“I think that the holistic approach (….) for me is like so important in this situation and keeping that communication and family involvement. Obviously if they can sense that and everything, I think that’s the main thing from that.”*

(Nurse, UK)

HCPs in the UK further noted that effective communication and information-sharing are essential for resolving issues related to sedation and symptom management. However, HCPs in the UK noted that some moral dilemmas cannot be completely resolved, leading them to rely on their personal moral codes.

Participants from Belgium emphasized the importance of team dialogue, including all caregivers, including general practitioners, to achieve consensus within the HCP team. This unified approach is essential for communicating consistently with the family about the patient’s care.

A participant in a German setting emphasized the importance of communicating to relatives that they, too, can contribute to their loved one’s care during palliative sedation.

*“And first of all, you have to sensitize the relatives to the fact that the subconscious is very awake and perceives the environment and perceives them. And that even in such a comatose state they can very well do something for the relative, the father, or actually not do anything in terms of taking action, but the decisive thing is being there for him and that he also perceives this.”*

(Catholic priest, Germany)

Additionally, HCPs in Belgium and Germany highlighted the significance of providing opportunities for family members to *say goodbye* to the patient.

*“…, but also, to give them the opportunity to get in touch with their loved ones and to give them the opportunity to say goodbye.”*

(Social worker 2, Germany)

In Hungary, the perceived lack of “good clinical practice” in communication about palliative sedation was viewed as a significant issue. Hungarian HCPs considered the absence of direct communication with patients a moral problem, as team members often spoke with family members first.

*“For me it’s always a dilemma, knowing that there are other practices in the world. In Hungary we somehow find, and many of us follow this, that we don’t directly sit down with the patient, it’s not in practice here, and we don’t tell them, look, you have months left and you’re going to die. But we always insist that the patient’s family member should be fully informed. I wonder if it is right to do that to the patient.”*

(Doctor 2, Hungary)

This approach was quite different from settings that put a larger emphasis on the principle of respecting patient autonomy, a value that was emphasized by HCPs in The Netherlands.

*‘In palliative care we always say that we develop care plans in coordination with the patient and the relatives, and considering their wishes and expectations, and it is stated as such in the definition of palliative care. And (….) yes the autonomy is a very important aspect in palliative care.’*

(Nurse 1, The Netherlands)

In Belgium, Italy, and Romania, the importance of seeking external support and advice in challenging cases was highlighted. The presence of updated guidelines and recommendations regarding palliative sedation and their communication was also seen as a crucial factor in addressing and ethically managing difficult cases.

### 3.2. Communication Issues Specific to Interactions Between Healthcare Professionals and Patients

As noted earlier, all HCP teams agreed that effective communication is fundamental to building trust and fostering collaboration with patients. However, achieving this requires HCP team members to actively invest in communication and trust-building, as this connection does not automatically exist from the outset. Poor communication with patients was felt to lead to a loss of trust, which may have detrimental effects on the overall treatment and care process.

HCPs in Germany, The Netherlands, Belgium, and Spain emphasized the importance of initiating timely communication about end-of-life (EOL) wishes. They stressed listening to the patient’s wishes while they are still physically and mentally able to express them and respecting these wishes as crucial elements of care.

*“I think if you can no longer communicate with someone, and that person suffers visibly, it would be a medical decision, as long as the criteria are met. But if you can still communicate with someone, then it is very important that you check the level of suffering in a conversation and whether someone is capable to say that they cannot or do not want to go on like this. Once they cannot communicate that anymore, that’s a problem, you would have to go on other things.”*

(Doctor, The Netherlands)

HCPs in the UK added that communication should also involve gathering as much nonverbal and indirect information as possible in situations where verbal or direct communication is not feasible.

*“I usually say I’m being a nosey nurse this visit and then after that I won’t be so nosey. We need to find out basically everything from their job—the patient picture really, so from the family—not the dynamics as such, but family, job—obviously there’s certain things like British, white or Muslim or whatever but we do try and get—obviously for me because I think faith is important or spirituality is very important, trying to get a spiritual—everything like that, the very personal things, what they did for jobs because you can obviously see with people whether it may be industrial but also, you get a personality of the patient. For instance, if they’re an engineer, you know that they’re a certain type of person. It’s things like that really so you get an idea, family dynamics, trying to get as much information as you can…”*

(Clinical Nurse Specialist, UK)

HCPs in Romania mentioned another issue: the difficulty of communicating openly with patients who are either unaware of their terminal condition or *unwilling to discuss* it.

*“ She [patient] communicated very little, didn’t agree to discuss preferences and prognosis. She avoided deeper discussions.”*

(Doctor 5, Romania)

Hungarian HCPs identified the approach of selective disclosure as a problem. Typically, they only inform patients about the subsequent steps of therapy, while full, open communication is directed towards the family members, including decisions about palliative sedation. Despite the HCPs’ limited communication, patients often become informed through other sources.

*“What you have to know is that the basis, or one of the bases, of our ostrich strategy is that patients are informed, they actually obtain information independently of us, a lot of information.”*

(Doctor 2, Hungary)

### 3.3. Communication Issues Specific to Interactions Between Healthcare Professionals and the Family

HCP teams across all participating countries agreed that communication about palliative sedation with the family should be a dialogue—a two-way process where HCPs both give and receive information. They also emphasized that this communication should be a unified team effort, ensuring that all relevant family members are informed, with the patient’s consent.

The importance of communicating with family members *at least as much* as with patients was emphasized in the UK and Italy.

*“And the function of the best interest meeting is to gather as much information before that decision really and that guides the decision doesn’t it along with the family as well, so I think perhaps the daughters would feel more comfortable with all that information and being involved in that discussion about that information*.”

(Nurse 1, UK)

*“Sometimes we have to work more with families than with patient”*

(Nurse 3, Italy)

Belgian HCPs emphasized the need to provide *tailored care for the family*, recognizing that they are the ones who remain after the patient’s death.

*“The way the patient would die could have a long-term impact on the life of the son”*

(Nurse 3, Belgium)

HCP teams in The Netherlands and Germany stressed that palliative sedation is primarily a medical decision. They emphasized the importance of supporting families with information and emotional assistance to help them understand these medical decisions. However, HCPs should not place the responsibility for deciding about the patient on the family.

*“I think there is a difference between giving the family the responsibility, or the feeling of responsibility for the decision and having a conversation about that decision. So if the team agrees palliative sedation is indeed the final step in the process, then you include the relatives, in this case the daughters, and discuss this decision with them without giving them the responsibility for that decision. You include them in the rationale of the team and the outcome of it, and then in that conversation you could very well ask how they think their father would’ve thought about this. Does this match his way of life? Those are the conversations you need to have. Relaying responsibility is something else.”*

(Nurse Specialist, The Netherlands)

*“…we would offer them not to decide alone, but to share the decision. So, the attempt to relieve the burden on the relatives …”*

(Psycho-oncologist, Germany)

In Germany and Belgium, MCD participants described the HCPs’ role as ensuring that everyone is on board and aware of what is happening. They emphasized the importance of continually checking in with all parties involved.

*“…but that you take them on board. Yes, and of course I think that’s very important, yes. It can’t go over their heads. But there must not be the feeling that this is the decision of the relatives.”*

(Catholic priest, Germany)

HCPs in Spain and Hungary observed that communication with family members differs between doctors and nurses. Nurses often find themselves in the middle of communication between doctors and family members, as family members are felt by nurses to communicate differently with nurses compared to doctors.

*“But I think that also, in the end, between nursing and the medical team, sometimes communication can also be difficult, why, because you are in the middle and, in the end, they come to call you and at the beginning you try to explain that the patient is comfortable, that there is no frown, that there is no complaint, they come to call you, come to call you and in the end you need support, because many times you don’t have, like, the gown. Straight away. Sometimes they need reassurance from their doctor, from the person who knows them or whatever.”*

(Nurse 2, Spain)

Italian MCD participants highlighted additional difficulties in communicating with family members, including the use of appropriate *terminology*. They noted that terms related to palliative sedation can be frightening for family members.

*“Often, caregivers are scared about the words like ‘morphine’ and ‘palliative sedation’, and because of this they can’t put themselves in patient’s shoes. It is necessary to do a teamwork, centered on communication.”*

(Nurse 3, Italy)

Hungarian HCPs pointed out the challenges of communicating with individuals from different cultural and ethnic backgrounds, particularly with a specific large minority. They also noted difficulties in managing these large families from the minority, who may give stronger emotional reactions contrary to the rest of the Hungarian population, especially when very large families arrive at the hospital to inquire about a patient. HCPs expressed concern about being accused of discrimination, which leads them to avoid discussing these communication issues, resulting in a lack of solutions.

A family of 25 comes in… (Nurse 3, Hungary)

*…. Yes, I did not dare to bring this topic up… the aspects of different cultures, or aspects of different customs. He (another doctor’s name) was abroad for quite some time, and he partly identified with that kind of different approach you need to take, which is a bit difficult for me.”*

(Doctor 2, Hungary)

### 3.4. Communication Issues Specific to Interactions Between the Patient and Their Family

Most HCP teams noted that they were often unaware of the communication dynamics within families and recognized that communication problems can arise due to shifting family roles. They emphasized the importance of supporting families in these situations.

HCPs in a German setting noted that patients’ communication behavior may change as they distance themselves from others—partly to avoid being a burden, and partly due to a reduced need for social contact.

*“…or the motivation that she doesn’t want to be a burden, when that comes up, I always find it difficult … or she doesn’t want any more contact, so there’s already a lot of withdrawal…”*

(Psycho-oncologist, Germany)

Furthermore, a participant in a German setting highlighted the importance of recognizing potential changes in relationships and social roles between patients and their relatives as the disease progresses, in order to provide appropriate support—for example, when a child takes on the role of caregiver for a parent.

*“…that this is such a very strong role change that they are going through. … that they now suddenly have to take responsibility for this father who used to be so strong.”*

(Protestant pastor, Germany)

Furthermore, in Belgium, patients not only received care but also took on the role of caregiver by helping their son cope with the grief associated with the patient’s illness.

*“How I experienced it, she put her son (a minor) first at that moment. uhm I think that was something she did as a mother, she didn’t want her son to be burdened by the fact she was suffering.*

(Nurse, Belgium)

Other communication-related issues included a lack of communication within families, which can lead to tensions, especially when patients and families disagree, as noted by HCPs in The Netherlands and Spain.

*“I cannot violate an advance directive of an incapable patient, validated by the capable woman. Even though my criteria are different, and I see suffering in the patient, how can I avoid the harm of this choice? By being there and minimizing the harm through consensus and negotiation, taking advantage of the crucial role of the daughter. What steps should I take? Consider a family meeting, mother, daughter and team.”*

(Doctor 3, Spain)

In Romania, it was observed that families sometimes insist on keeping the patient able to communicate until the very end, which can delay the decision to initiate sedation.

*“There was a family meeting with the two daughters, but although they understand their father’s illness, it is very hard for them to accept that their father will be gone and … they wish, by the end of his life, to be able to communicate with him, not to be sedated.”*

The postponement of the decision makes them believe that he will stay with them.

(Nurse, Romania)

Italian HCPs highlighted the specific issue of informal (only oral, not written) delegation of responsibility to a family member, such as a husband, by the patient. They expressed concern that the family member may struggle to make the right decisions due to poor communication between the patient and the family member.

*“X (patient’s name) has always delegated her husband for choices that concerned her health; so we have this husband that isn’t convinced about palliative sedation because, obviously, he still hopes that she’ll recover. And he is the informal delegate of the patient.”*

(Doctor 1, Italy)

HCPs in the German settings and the UK emphasized that *understanding the relationships and communication dynamics* within the family is crucial for providing effective support.

*“…to sort of go back to another point about that sort of a different pace of grief or acceptance of the situation that their loved one’s dying and they’re not ready for that yet and sometimes that disagreement is caused by the different paces of people in the family*.”

(Nurse Specialist, United Kingdom)

## 4. Discussion

To our knowledge, this is the first study to examine HCPs’ perceptions of communication issues related to palliative sedation during MCD sessions across European countries. Given its novelty, direct comparisons with earlier research are limited. Although the findings cannot be generalized to national perspectives, they offer important insight into how HCPs perceive communication challenges. Our findings revealed that many challenges were common across sites, with the main difference surrounding the provision of information to patients. While there was consensus that patients have the right to know, in some countries, information was not always fully communicated. HCPs also noted that challenges varied depending on their personal experiences and cultural backgrounds.

Empathic, clear, open, and honest communication was deemed essential for establishing trust between the HCP team, patients, and family members, and this trust should be built from the initiation of palliative treatment. All HCP members agreed that both the patient and the family have the right to be informed, necessitating a continuous, two-way exchange of information. This process should be a team effort, carried out in a unified manner with the family, ensuring that the patient’s decisions and autonomy are fully respected. These objectives align with current communication guidelines for palliative sedation, which advocate for including patients, family members, and the healthcare team in the decision-making process to initiate proportionate palliative sedation [5,18,26,27,28].

Across all sites, the principle that patients and families have a right to information was recognized, yet the way it was enacted differed. In The Netherlands, communication was autonomy-centered, possibly supported by structured tools, which encourage patients to voice symptoms and thereby facilitate earlier, shared decisions about palliative sedation [29]. German and Belgian HCPs emphasized continuity and repeated dialogue with families [30,31]. Earlier German qualitative research has shown that early conversations, continuity, and systematic involvement of relatives are considered prerequisites for effective end-of-life communication [30], and a Belgian study on continuous deep sedation confirmed that nurses frequently acted as mediators, supporting families before and during sedation [31]. UK HCPs emphasized the importance of proactively inviting questions, aligning with previous UK research that underscores how communication strategies can support understanding and foster collaborative decision-making in end-of-life discussions [32]. By contrast, Romanian and Hungarian HCPs described reluctance to openly discuss dying, often prioritizing family over patient communication [33,34]. Previous analyses in Romania have highlighted structural weaknesses in palliative care and the challenges nurses face in maintaining patient dignity within resource-constrained systems [35,36], while Hungarian ICU and survey data highlight selective disclosure practices [34,37]. In Spain and Italy, HCPs noted the presence of a family-centered approach, where large family networks may complicate communication processes and lead to the informal delegation of decisions by the patient to family members. Earlier studies in Spain appear to support this, as they have indicated that families’ perceptions of accompaniment and person-centeredness strongly shape their acceptance of end-of-life decisions [38,39].

Maintaining open dialogue with patients and their loved ones throughout the palliative sedation process is fundamental [5,40]. HCPs in Germany, The Netherlands, Belgium, and Spain in our study emphasized the importance of listening to patients and initiating timely communication about their wishes while they are still physically and mentally capable. Respecting these wishes was deemed crucial, as poor communication can undermine trust and negatively affect the entire care process according to previous studies [27,41,42].

HCPs in the UK highlighted that effective communication also involves gathering non-verbal information in addition to verbal cues, as patients and families often need explicit invitations to share their views. This is important because recent studies have shown that patients and their family members often find it challenging to ask for additional information and tend to prefer a proactive approach by HCPs [42,43,44]. Conversations about palliative sedation should start when prognosis is still measured in months and continue as life expectancy shortens, including documentation of the patient’s decisions, consistent with revised EAPC guidelines [5,18].

In the UK and Italy, participants emphasized communicating with family members as much as with patients. Bruinsma et al. [40] found that family members are more likely to accept palliative sedation when sufficiently informed and involved. Belgian healthcare professionals emphasized the importance of providing tailored care to families, recognizing that they are the ones who remain after the patient’s death. This perspective aligns with family members’ requests for healthcare professionals to provide personalized support and to acknowledge their roles as caregivers [44,45,46].

Shifting family roles emerged as a unique challenge. In Germany, healthcare professionals observed that some patients may withdraw from their family members as their communication behavior shifts. This distancing was understood as a way for patients to avoid burdening others and a reflection of their decreasing need for social interaction. For instance, in Belgium, patients often took on the role of caregiver themselves, supporting their children as they navigated the grief associated with the patient’s illness. This observation aligns with recent findings that some patients prefer to shield their family members from the details of their condition, fearing the added burden and anxiety this information may cause [47].

Understanding family relationships and communication dynamics can be crucial in these situations, as noted by HCPs in Germany and the UK, to provide the best support. Additionally, HCPs in The Netherlands and Germany highlighted the importance of assisting families in their decision-making by providing information and emotional support, while clarifying that the responsibility for medical decisions rests primarily with the healthcare team. This approach ensures that families are informed without placing undue pressure on them to decide about the patient’s care.

Romanian HCPs reported difficulty openly discussing dying, often due to patients’ reluctance or unawareness of their condition. Contextual barriers, including limited resources and the insufficient integration of palliative care, further constrain communication between healthcare providers and patients [33,35]. However, this communication challenge has also been noted in previous studies, where patients frequently find it difficult to cope with information about their declining health and impending end of life [26,27,48]. In certain instances, the patient might prefer to avoid discussing end-of-life issues and palliative sedation, often choosing to delegate this responsibility to a family member [5]. Such informal delegation of decisions to family members can create stress for the team, particularly if this informal delegation results in decisions that the HCP team believes prolong the patient’s suffering, as noted by Italian HCPs.

Selective disclosure was a major communication issue in Hungary. Hungarian HCPs often communicated first with family members, and patients were informed primarily only about subsequent steps of their treatment rather than being engaged in open discussions throughout the process. While informing the patient first is standard in Northern Europe and North America, studies indicate this approach may be less common elsewhere [37]. Differences in how much patients and families are informed may partly reflect declining patient decision-making capacity near the end of life. However, as also noted in our study, in some settings, fewer patients were informed than their family members—a disparity that likely cannot be attributed to the patient’s medical condition alone. Family members often seek more information than patients [48,49], and as end-of-life approaches, they value opportunities to resolve issues among themselves and say their goodbyes [45,46]. Consistent with patient and family needs, healthcare providers in Belgium and Germany emphasized facilitating and encouraging saying goodbye.

Nurses were consistently identified as critical mediators within the palliative team, facilitating communication with family members and bridging gaps between patients and physicians. For example, a study in The Netherlands found that, unlike physicians—who were present only 50% of the time during palliative sedation—nearly all palliative care nurses were present from the decision-making phase through to the conclusion of the procedure [50]. Our findings from Hungary and Spain similarly indicated differences in communication styles between doctors and nurses: nurses often received more or different information from patients and families, likely due to spending more time with them, and relayed relevant details to the physicians. This intermediary role can place nurses in challenging positions, as they must maintain the trust of both the doctor and the patient (and family), while navigating confidentiality constraints [37]. Multiple studies corroborate that nurses, being most deeply involved in direct patient care, regularly experience moral distress when communicating about or administering palliative sedation. For instance, UK hospice nurses reported ethical dilemmas such as feeling compelled to act contrary to what they believed was best—whether by rushing sedation or responding to family requests they found ethically challenging [51]. Global reviews similarly highlight that nurses face unique challenges in communication, regarding ethical issues in decision-making, and reconciling limited choices with their moral responsibility to do no harm [9,52]. These findings underscore the importance of supporting nurses through communication training and ethical reflection to reduce moral distress and improve care outcomes.

Terminology surrounding palliative sedation was identified as a source of misunderstanding, indicating the need for the use of clear, standardized language. Earlier research has shown that the general public—and occasionally even medical professionals—often perceive continuous palliative sedation as a form of physician-assisted suicide or euthanasia [53,54]. Therefore, it is clear that providing detailed and explicit information is essential before end-of-life events. Italian healthcare providers noted that many family members found the term frightening, highlighting the need to address this issue. Providing detailed information before end-of-life events allows for shared decision-making, consistent with the three-talk model [55]. Evidence shows that unclear communication can increase emotional distress among family members [56].

Seeking advice from external supervising support can be beneficial in challenging cases of palliative sedation. Additionally, having updated and implemented guidelines and recommendations can help facilitate communication and effectively manage these complex situations. However, because the practice of palliative sedation varies across countries due to cultural differences and diverse healthcare systems, only general guidelines and recommendations can be provided for communicating about palliative sedation [18,55]. For instance, Hungarian healthcare providers noted difficulties in communicating with members of a large minority group, whose families often exhibit different emotional reactions compared to the broader Hungarian population. They lack specific recommendations for these interactions and hesitate to address the issue outside the team for fear of being accused of discriminatory language.

## 5. Limitations

Our study has several limitations. First, MCDs were conducted in different languages across countries, so some nuances may have been lost in translation to English. Second, while some HCPs were familiar with the MCD process, others were not, which could have influenced how openly they expressed their views. Third, although the sample size was fairly large for a qualitative study, the number of participants and sites per country was relatively small, limiting generalizability. We did not systematically collect information on participants’ or facilities’ religious affiliations, which may have influenced perspectives on communication around palliative sedation in some settings.

Additionally, this study was exclusively qualitative. Complementary quantitative research could further elucidate healthcare professionals’ experiences of ethical dilemmas and provide a broader, more generalizable understanding. Similarly, our focus was primarily on communication with patients and their families; perspectives from other professional interactions were not explored, which may limit the scope of the findings regarding interdisciplinary communication and point to an area for future investigation.

Despite these limitations, several strengths should be noted. This study addresses a gap in the literature, as no prior studies have examined this specific topic. The large number of participating healthcare professionals and the interdisciplinary composition of the teams provide a wide range of insights into communication in palliative sedation. Moreover, the MCD method proved highly beneficial, especially for teams previously unfamiliar with it, supporting its potential integration into clinical practice.

## 6. Conclusions

Our study provides a perspective on healthcare professionals’ perceptions of communication challenges in palliative sedation across eight European countries. While empathic, clear, and continuous communication was universally recognized as essential, the ways in which patients and families are informed differed, possibly reflecting both cultural norms and systemic factors.

Some tendencies were observed across sites. For example, participants from North-Western European countries more often described autonomy-centered practices that emphasized timely patient discussions, ongoing family dialogue, and proactive invitation of questions. In Southern and Eastern European sites, family-centered communication appeared more prominent, sometimes involving selective disclosure to patients or initial delegation of decision-making to relatives. These observations should not be interpreted as nationally representative but rather as illustrative of the cases and professionals included. Certain communication challenges—such as informal decision-making delegation in Italy, shifting family dynamics in Germany, and reluctance to discuss dying according to Romanian HCPs—appeared less tied to national norms and more to individual professional experiences or specific cases. Across all countries, nurses played a key mediating role, bridging gaps between patients, families, and physicians, while terminology around palliative sedation remained a source of misunderstanding.

Contextual barriers—including resource constraints, limited integration of palliative care, and cultural diversity—shaped communication practices. Addressing these challenges requires structural support and targeted education. Initiatives such as undergraduate and postgraduate curricula, workplace-based training, and reflective formats (e.g., ethics rounds, case-based discussions, or interprofessional workshops) can help healthcare professionals navigate ethically complex situations, reconcile moral dilemmas, and engage patients and families effectively. To further support resolution of dilemmas that cannot be fully addressed in practice, we recommend integrating ethics simulations, structured consultation services, and reflective interprofessional workshops. These interventions provide safe spaces for discussion, reduce reliance on individual moral codes, and promote shared decision-making.

Our study highlights the importance of dialogue among patients, families, and healthcare professionals, and the need for communication training in settings with limited provider skills. Although guidelines exist, implementation is often inconsistent, emphasizing the need for culturally tailored protocols. Policy efforts should embed communication training within palliative care programs, develop standardized yet adaptable protocols, and support interdisciplinary reflection at national and institutional levels. Further research is needed to evaluate these interventions and deepen the understanding of communication challenges across diverse cultural and clinical contexts.

## Figures and Tables

**Table 1:** Characteristics of the clinical sites and participants in the MCD sessions.

Country	Clinical Site		N^o^ of Participants	Profession of the Participants
1. Belgium	1. Department of Palliative Care	Session 1	6	1 physician, 2 nurse specialists, 1 spiritual counselor, 1 psychologist, 1 social worker
		Session 2	6	1 physician, 1 psychologist, 1 social worker, 2 nurse specialists, 1 spiritual counselor
	2. Department of Palliative Care	Session 1	6	3 nurse specialists, 1 nurse, 2 physicians
		Session 2	6	2 physicians, 3 nurse specialists, 1 nurse
2. Germany	3. Department of Palliative Medicine	Session 1	7	1 catholic priest, 2 nurses, 1 physician, 1 protestant pastor, 1 psychologist, 1 social worker
		Session 2	8	1 catholic priest, 5 nurses, 2 physicians
	4. Centre for Palliative Medicine	Session 1	3	1 nurse, 1 physician, 1 social worker
		Session 2	5	2 nurses, 1 physician, 1 psycho-oncologist, 1 social worker
3. Hungary	5. Department of Neurosurgery	Session 1	9	4 physicians, 4 nurses, 1 pharmaceutical assistant
		Session 2	6	2 physicians, 4 nurses
	6. Department of Otorhinolaryngology and Head and Neck Surgery	Session 1	9	4 nurses, 5 physicians
		Session 2	7	4 physicians, 3 nurses
4. Italy	7. Pain therapy and supportive care unit	Session 1	9	5 nurses, 3 healthcare assistants, 1 physician
		Session 2	8	4 nurses, 3 healthcare assistants, 1 physician
	8. Hospice	Session 1	8	4 nurses, 2 healthcare assistants, 2 physicians
		Session 2	7	2 nurses, 2 healthcare assistants, 3 physicians
5. The Netherlands	9. Department of Anesthesiology, Pain and Palliative Care	Session 1	9	5 physicians, 2 nurse specialists, 2 spiritual counselors
		Session 2	6	4 physicians and 2 nurse specialists
	10. Oncology department	Session 1	9	3 physicians, 4 nurse specialists, 2 spiritual counselors
		Session 2	7	1 physician, 3 nurse specialists, 3 spiritual counselors
6. Romania	11. Hospice Casa Sperantei 1	Session 1	7	1 physician, 3 nurses, 2 social workers, 1 psychologist
		Session 2	7	1 physician, 3 nurse specialists, 3 spiritual counselors
	12. Hospice Casa Sperantei 2	Session 1	8	4 physicians, 1 nurse, 1 social worker, 1 psychologist, 1 spiritual counselor
		Session 2	8	4 physicians, 1 nurse, 1 social worker, 1 psychologist, 1 spiritual counselor
7. Spain	13. Department of Palliative Care and Department of Oncology	Session 1	9	4 physicians, 4 nurse specialists, 1 psychologist
		Session 2	6	2 physicians, 3 nurse specialists, 1 psychologist
	14. Department of Palliative Care and Department of Oncology	Session 1	9	4 physicians, 5 nurse specialists
		Session 2	9	4 physicians, 5 nurse specialists
8. United Kingdom	15. Hospice Inpatient Unit 1	Session 1	5	1 social worker, 1 nurse, 2 advanced nurse practitioners, 1 nurse specialist
		Session 2	5	1 medical director, 1 social worker, 1 nurse, 1 nurse specialist, 1 advanced nurse practitioner
	16. Hospice Inpatient Unit 2	Session 1	9	4 nurse specialists, 1 chaplain, 1 healthcare assistant, 1 nurse, 1 medical director, 1 physician
		Session 2	6	2 nurse specialists, 1 chaplain, 1 nurse, 1 medical director, 1 physician

**Table 2:** Main Categories and key communication issues on palliative sedation from MCDs in 16 clinical settings across eight European countries.

Main Category		Key Communication Issues
General issues in communication		- Communicating with empathy, clarity, and openness - Tailoring communication to individual patients - Ensuring continuity and flow of information - Supporting patients and families in saying goodbye - Seeking external communication support for HCPs - Lack of or need for updated guidelines on communication
Communication issues specific to interactions between:		
	Healthcare professionals—Patient	- Listening to and respecting the patient’s wishes - Gathering direct and indirect information from patients - Patients’ unwillingness or inability to discuss end-of-life topics
	Healthcare professionals—Family	- Recognizing communication as a two-way process - Providing tailored support and information to families - Communication challenges with families from diverse cultural or ethnic backgrounds - Nurses experiencing tension or role conflict when mediating between patients, families, and physicians
	Patient- Family members	- Shifting roles and responsibilities within families - Patient’s fear of overburdening family members - Tensions caused by a lack of intra-family communication - Informal transfer of decision-making to family members

## Data Availability

The datasets used and/or analyzed during the current study are available from the corresponding author upon reasonable request.

## Abbreviations

CanMeds	Canadian Medical Education Directive for Specialists
EAPC	European Association for Palliative Care
EOL	End-of-life
HCP	Healthcare professional
MCD	Moral case deliberation
PS	Palliative sedation
UK	United Kingdom
